# Development of a general logistic model for disease risk prediction using multiple SNPs

**DOI:** 10.1002/2211-5463.12722

**Published:** 2019-09-27

**Authors:** Cheng Long, Guanting Lv, Xinmiao Fu

**Affiliations:** ^1^ West China Hospital of Sichuan University Chengdu Sichuan China; ^2^ Beijing Institute of Genomics Chinese Academy of Sciences Beijing China; ^3^ Provincial University Key Laboratory of Cellular Stress Response and Metabolic Regulation College of Life Sciences Fujian Normal University Fuzhou Fujian China

**Keywords:** disease risk prediction, GWASs, logistic regression, personalized medicine, precise medicine, SNP

## Abstract

Human diseases are usually linked to multiloci genetic alterations, including single‐nucleotide polymorphisms (SNPs). Methods to use these SNPs for disease risk prediction (DRP) are of clinical interest. DRP algorithms explored by commercial companies to date have tended to be complex and led to controversial prediction results. Here, we present a general approach for establishing a logistic model‐based DRP algorithm, in which multiple SNP risk factors from different publications are directly used. In particular, the coefficient β of each SNP is set as the natural logarithm of the reported odds ratio, and the constant coefficient β_0_ is comprehensively determined by the coefficient and frequency of each SNP and the average disease risk in populations. Furthermore, homozygous SNP is considered a dummy variable, and the SNPs are updated (addition, deletion and modification) if necessary. Importantly, we validated this algorithm as a proof of concept: two patients with lung cancer were identified as the maximum risk cases from 57 Chinese individuals. Our logistic model‐based DRP algorithm is apparently more intuitive and self‐evident than the algorithms explored by commercial companies, and it may facilitate DRP commercialization in the era of personalized medicine.

**Abbreviations**
DRP: disease risk prediction
GWAS: genome‐wide association study
OR: odds ratio
SNP: single‐nucleotide polymorphism

Genome‐wide association studies (GWASs) are increasingly uncovering the effect of genetic alterations in most of the common diseases 1 and are considerably accelerating the commercialization of personalized medicine 2. One of the genetic alteration based applications for personalized medicine is disease risk prediction (DRP) 3, 4, 5, which has been reported to benefit individuals in terms of improving their lifestyle choices and facilitating preventive screening 3, 4, 6, 7. However, the discrepancy among DRPs of different direct‐to‐customer companies (e.g., 23andMe, Navigenics and deCODEme) has been widely reported 8, 9, 10, 11 and substantially limits their commercialization. Such discrepancy has been mainly attributed to the difference in the selection of single‐nucleotide polymorphism (SNP) risk factors and DRP algorithms. Although it is impossible for different DRP providers to select a palette of the same SNP risk factor markers for one type of disease, there is a high demand for the development of a general DRP algorithm.

Retrospectively, the algorithms of three DRP companies (23andMe, deCODEme and Navigenics) have notable differences in principle (as described in Data S1) and are complicated and compromised 9, 10. Whereas 23andMe and deCODEme transform the odds ratio (OR) of each SNP into a likelihood ratio, Navigenics transforms it into the relative risk. Thereafter, 23andMe multiplies the likelihood ratios of single genotypes by the average odds of the disease and converts these odds into risks, deCODEme multiplies the likelihood ratios by the average risk of disease, and Navigenics calculates the relative risks for all of the possible genotype combinations.

Apparently, these DRP algorithms are not suitable for generalization because of the following limitations 9, 10. First, all of them require the OR values of both homozygous and heterozygous SNPs. However, the OR values for many homozygous SNPs are not available in the existing GWAS literature, and this may limit the utility of high‐risk SNPs whose OR values are available for only the heterozygous forms. Second, the DRP algorithm used by Navigenics requires a substantial amount of computer working memory 10. Third, the DRP algorithm used by deCODEme is apparently not normalized; that is, the calculated risk might be larger than the upper limit value of 100% under certain circumstances. It is thus highly demanding to develop a more self‐evident, robust and reliable algorithm for DRP.

Logistic regression has been widely used for risk factor identification and evaluation in general, 12 as well as in GWASs in particular. After a regression analysis of the experimental data, the generated logistic model containing certain risk factors can be applied for DRP 5, 12, 13, 14, 15. However, the SNP risk factors related to a disease are usually identified in many studies by different groups of researchers; therefore, the generated logistic models in these studies are also different and would not be suitable for DRP directly. From this perspective, a method to build a general logistic model that integrates multiple SNP risk factors from multiple publications is in high demand for DRP. The algorithms explored by commercial DRP companies represent such efforts but apparently cannot meet the needs 8, 9, 10, 11.

Here, we present a general approach to build a logistic model for DRP, in which multiple SNPs from multiple publications are integrated and can be promptly updated. Our model largely overcomes the aforementioned limitations of the currently used algorithms. Furthermore, we validated the model on lung cancer with Chinese individuals, illustrating its significance in preventive screening.

## Materials and methods

### Data collection from the literature

For each selected SNP, the original literature was downloaded, and the OR values for both homozygous (if available) and heterozygous SNPs were collected. SNPs with an OR <1.15 were omitted. Then, the frequency of each SNP in Chinese individuals and all individuals was extracted from the National Center for Biotechnology Information database by searching each SNP ID (for details, refer to Table 1), and the lifetime risk for lung cancer in Chinese men and women was assigned according to the Chinese Cancer Registry Annual Report 2010 16. These data were used by the algorithm for the regression analysis, as described in the Results and Discussion.

**Table 1:** SNPs and parameters of logistic model for lifetime risk prediction of lung cancer. NA, not available.

SNP ID	Risk base	Frequency in Chinese individuals	Frequency in all individuals	OR of heterozygous SNPs	OR of homozygous SNPs	References	aβ	bβ_0_ calculation
rs1820453	G	0.239	0.374	1.49	1.65	20	0.399	0.174
rs716274	G	0.25	0.438	1.83	2.96	20	0.604	0.294
rs9981861	G	0.125	0.332	1.33	cNA	21	0.285	0.036
rs16951095	C	0.805	0.917	1.3	NA	22	0.262	0.211
rs1051730	T	0.037	0.185	1.31	NA	23	0.270	0.010
rs402710	C	0.733	0.657	1.18	NA	24	0.166	0.121
rs2808630	G	0.22	0.211	1.22	NA	25	0.199	0.044
rs7626795	G	0.207	0.246	1.16	NA	25	0.148	0.031
Men	cRisk	β_0_	Women	cRisk	β_0_		Sum	0.921
	0.0562	−3.742		0.0256	−4.560			

^a^In the logistic model, coefficient β for each SNP was calculated as the natural logarithm of the OR value of the heterologous SNP on the basis of Eq (3). ^b^For SNP without the OR value of the homozygous form, it was the product of β and the frequency in Chinese individuals (e.g., for rs9981861, 0.285 × 0.125 = 0.036). Otherwise, it was calculated as the sum of two products contributed, respectively, by the heterozygous and homozygous SNPs [e.g., for rs1820453, according to the Hardy‐Weinberg equilibrium, the frequencies of heterozygous and homozygous SNPs in Chinese individuals were calculated as 2 × 0.239 × (1 − 0.239) = 0.364 and 0.239 × 0.239 = 0.057, respectively, and the total contribution was calculated as ln(1.49) × 0.364 + ln(1.65) × 0.057 = 0.174]. ^c^The average lifetime risk for lung cancer in Chinese men and women was set as 5.62% and 2.56%, respectively, according to the Chinese Cancer Registry Annual Report 2010 16.

### Ethics approval and consent to participate

The experiments in genotyping human SNPs were approved by the Ethics Committee of Third Hospital of Peking University. We confirm that our study is compliant with the ‘Guidance of the Ministry of Science and Technology of China for the Review and Approval of Human Genetic Resources’, and the study methodologies conformed to the standards set by the Declaration of Helsinki. The experiments were undertaken with the understanding and written consent of each subject.

### Human subjects

Patients were normally subjected to different clinical tests, including peripheral blood‐based clinical tests. The remaining blood (around at a volume of 1.5 mL) of each patient, accompanied with the information of only sex, age, smoking and clinical symptoms, was transferred by the hospital to us for DNA extraction and SNP genotyping, with the data being analyzed anonymously.

### SNP genotyping

Genomic DNA was extracted from peripheral blood by using a QIAamp DNA Blood Mini Kit (Qiagen, Valencia, CA, USA) in accordance with the manufacturer's instructions. The primers for SNP genotyping were designed using MassArray Assay Design v4.0 according to the sequence information flanking the SNP, were synthesized by Invitrogen and were validated by MassArray Analyzer Compact. The PCR, removal of free dNTP and sequencing by MassArray Analyzer Compact (Sequenom, San Diego, CA, USA) were performed according to the manufacturer's instructions.

### Statistics

Each sample was resequenced independently, and all of the SNP sequencing results were consistent between independent repeated experiments. Risk calculation was performed using Microsoft Excel (Microsoft, Redmond, WA, USA). A statistical analysis was performed in the microorigin software using the ANOVA algorithm at a significance level of 0.05.

## Results and Discussion

### Logistic model generated from a designed study does not meet the need for DRP on an increasing number of SNP‐related diseases

Logistic regression has been widely used for risk factor identification and evaluation, and is generally presented as follows:

(1) $$\text{Logit}\left(P\right)=\text{ln}\left(\frac{P}{1-P}\right)=\beta_{0}+\beta_{1}x_{1}+\beta_{i}x_{i}\ldots +\beta_{n}x_{n}$$

where β_*i*_ and *x*_*i*_ are the coefficient and the logic value of each risk factor, respectively. Then, the disease risk *P* is expressed as follows:

(2) $$P=\frac{1}{1+e^{-(\beta_{0}+\beta_{1}x_{1}+\beta_{i}x_{i}\ldots +\beta_{n}x_{n})}}.$$

In a research study, researchers usually use a majority of their experimental data to estimate the coefficients in Eqns (1,2) by using statistical software through a maximum likelihood estimation algorithm 12. Then, the remaining experimental data are subjected to the generated logistic model for validation 12. Further, researchers can use the generalized model to predict the disease risk of new subjects. Apparently, this strategy is useful and effective for DRP based on the traditional risk factors 12.

In general, only one or a few novel SNP risk factors related to a disease are identified in a specific GWAS, in which the coefficients are determined and a specific logistic model is generated as described above. If the disease is linked to only these SNP risk factors, the logistic model generated from this GWAS is suitable for DRP directly. However, most human diseases are usually linked to a large number of SNP risk factors (sometimes up to 100 17), and each of them has a minor effect on the disease risk. In particular, these SNPs are identified one by one by different research groups and are thus timely reported in multiple publications. Accordingly, the logistic models generated in these studies are different and are not interchangeable for DRP directly.

### Generation of the logistic model for DRP by directly using multiple SNPs from multiple publications

Now, the question is how to integrate the multiple SNPs from multiple publications to build a general model for DRP. Commercial DRP companies have developed algorithms to directly use the information of multiple SNPs from multiple publications for DRP by incorporating the OR value and the population frequency of each SNP, which are usually available in the GWAS literature and/or public databases. These algorithms, however, are complicated and lead to discrepant DRP results 8, 9, 10, 11. We thought to integrate the multiple SNP risk factors from multiple publications into a logistic model for DRP (as indicated by Eqn 2).

Regarding Eqn 2 for DRP, we need the coefficient β_*i*_ of each SNP and the constant coefficient β_0_, as well as the value of *x*_*i*_ for each SNP that can be determined by the SNP genotyping of the subject. One way to determine β_*i*_ and β_0_ is to resequence the known SNP risk factors in a large number of subjects and then perform a logistic regression analysis. This strategy, although used previously 5, does not make full use of the knowledge of the SNP risk factors that have been reported and thus has compromised cost‐effectiveness. In particular, if a new SNP risk factor related to the disease is discovered, an additional clinical study needs to be performed for its incorporation into the logistic model.

In our approach, β_*i*_ in Eqn ( 2) is not estimated by a statistical analysis on the clinical data as performed by Ripatti *et al*. 5. Rather, it is obtained directly by transforming the reported OR value of the SNP according to the logistic regression as follows:

(3) $$\beta_{i}=\text{ln}\left({\text{OR}}_{i}\right)$$

This is based on the definition of OR in a logistic regression model as follows:

(4) $${\text{OR}}_{i}=\frac{\text{odds}(x_{i}+1)}{\text{odds}(x_{i})}=\frac{\frac{P(x_{i}+1)}{1-P(x_{i}+1)}}{\frac{P(x_{i})}{1-P(x_{i})}}=\frac{e^{\beta_{0}+\beta_{1}x_{1}+\beta_{i}x_{i}+\beta_{i}+\ldots +\beta_{n}x_{n}}}{e^{\beta_{0}+\beta_{1}x_{1}+\beta_{i}x_{i}+\ldots +\beta_{n}x_{n}}}=e^{\beta_{i}}$$

where the OR value of a SNP risk factor is explained as the ratio of the relative disease risk of the person at risk to the relative disease risk of a person not at risk.

Next, β_0_ can be determined as follows: if a SNP risk factor plays a role in the disease development, its contribution with respect to individuals should be considered the same as that with respect to populations. Then, Eqn 1 for an individual can be converted to the following form for populations:

(5) $$\text{ln}\left(\frac{P_{0}}{1-P_{0}}\right)=\beta_{0}+\beta_{1}f_{1}+\beta_{i}f_{i}\ldots +\beta_{n}f_{n}$$

where the population frequency (*f*_*i*_) of each SNP and the average population disease risk (*P*_0_) are usually available in public databases; the coefficient (β_*i*_) of each SNP has been already determined through Eqn 3. Then, β_0_ can be calculated using Eqn 6 as follows:

(6) $$\beta_{0}=\text{ln}\left(\frac{P_{0}}{1-P_{0}}\right)-\left(\beta_{1}f_{1}+\beta_{i}f_{i}\ldots +\beta_{n}f_{n}\right)$$

Apparently, β_0_ reflects the contribution of the unidentified and/or unselected SNP risk factors, as well as of the non‐SNP risk factors, including the traditional risk factors (e.g., smoking and drinking). Importantly, if new critical SNPs are identified and need to be incorporated into our model, β_0_ can be recalculated according to Eqn (6). In other words, our logistic model for DRP can be updated quickly if necessary.

In addition, the variance of *P* for each subject is collectively determined by the sum of the product of the variance of each β_*i*_ and the logic value *x*_*i*_ (according to Eqn 2), with the variance of each β_*i*_ being derived from the variance of OR for each SNP (according to Eqn 3). In this regard, OR with a smaller variance is favorable when multiple ORs are available for a specific SNP as reported in multiple publications.

Equations (1), (2), (3), (4), (5), (6) represent a general approach to establishing a logistic model‐based DRP algorithm by combining multiple SNPs from multiple publications, as summarized here. First, the SNP risk factor markers for a specific disease are selected from the existing GWAS literature, collecting their OR values and population frequencies. Second, the coefficient β_*i*_ of each SNP and the constant coefficient β_0_ are determined using Eqns 3 and 6, respectively; therefore, Eqn 2 for DRP is ready. Third, the SNP risk factors for the subject are determined experimentally. Fourth, DRP is performed using Eqn (2). Note that both homozygous and heterozygous SNPs are suitable for DRP in our algorithm. If the OR value for the homozygous form of a SNP is available, it is considered a dummy variable and *x*_*i*_ is set as ln(OR_homozygous_)/ln(OR_heterozygous_).

### Collection of SNP markers for lung cancer risk prediction in Chinese individuals

Thus far, about 400 000 reference SNPs have been deposited for human genomes (refer to the National Center for Biotechnology Information SNP database: https://www.ncbi.nlm.nih.gov/snp/docs/RefSNP_about/). Of these, about 71 600 SNPs have been found to be associated with human diseases and traits, as reported in the NHGRI‐EBI GWAS Catalog database 18, 19 ( https://www.ebi.ac.uk/gwas/). Lung cancer is considered a partially inherited complex disease 4, and about 80 SNPs have been reported to be associated with it 17. Among these SNPs, a threshold value of 1.15 was set subjectively, and some genetically dependent SNPs were omitted. In the end, eight representative SNPs were selected for the lung cancer risk prediction in our study. The OR value and the population frequency for each of these eight SNPs in Chinese individuals were collected (Table 1).

### Generation of algorithms for lung cancer risk prediction

Then, we determined β for each SNP (Table 1) according to the aforementioned rules. Given the lifetime risk for lung cancer in Chinese men and women being 5.62% and 2.56%, respectively, β_0_ was calculated as −3.742 and −4.560 (for details, refer to Table 1). Together, the SNP‐based risk prediction for lung cancer with Chinese men and women was finalized as follows:

(7) $$P\left(\text{men}\right)=1/\left(1+e^{3.742-0.399x_{1}-0.604x_{2}-0.285x_{3}-0.262x_{4}-0.270x_{5}-0.166x_{6}-0.199x_{7}-0.148x_{8}}\right)$$

(8) $$P\left(\text{women}\right)=1/\left(1+e^{4.560-0.399x_{1}-0.604x_{2}-0.285x_{3}-0.262x_{4}-0.270x_{5}-0.166x_{6}-0.199x_{7}-0.148x_{8}}\right)$$

### Utilization of the algorithm for lung cancer risk prediction in Chinese individuals

To demonstrate the effectiveness of our logistic algorithm, we collected blood samples of 48 Chinese subjects (38 men and 10 women), genotyped the eight selected SNPs (for details, refer to Table S1) and then calculated the lung cancer risk of each subject by using Eqns (7,8). Remarkably, subject 17, who was diagnosed as the sole lung cancer patient among these 48 subjects and had the symptoms of poorly differentiated adenocarcinoma in the right lung and multiple metastases, had the highest absolute lifetime risk of 28.3% and a relative lifetime risk of 5.0 (Table 2). The absolute lifetime risk of this patient was significantly higher than that of the remaining 47 persons (*P *=* *0.0000024) and was significantly higher than that of the remaining 37 men (*P *=* *0.000015). In addition, the average lifetime risk for the 38 men and the 10 women was 8.9% and 5.5%, respectively (Table 2); both of these values were not significantly different from the average disease risk of men and women (*P *>* *0.05).

**Table 2:** Lung cancer risk and clinical symptoms for 48 subjects.

ID	Absolute risk	Relative risk	Sex	Symptom
17	0.283	5.0	M	Poorly differentiated adenocarcinoma in the right lung, multiple metastases
23	0.174	3.1	M	Normal
27	0.174	3.1	M	Normal
25	0.152	2.7	M	Normal
26	0.145	2.6	M	Normal
16	0.141	2.5	M	Normal
2	0.138	2.5	M	After surgery for the right knee ligament reconstruction
32	0.122	2.2	M	Normal
22	0.119	2.1	M	Normal
24	0.119	2.1	M	Normal
19	0.094	1.7	M	Normal
4	0.092	1.6	M	Lumbar spinal stenosis
10	0.092	1.6	M	Lumbar disc herniation
11	0.092	1.6	M	Normal
30	0.087	1.5	M	Normal
28	0.086	3.4	F	Non‐Hodgkin's lymphoma
15	0.084	1.5	M	Normal
43	0.081	1.4	M	Normal
18	0.081	3.2	F	Tubal pregnancy
33	0.078	1.4	M	Normal
8	0.077	1.4	M	Normal
31	0.077	3.0	F	Cholecystitis
9	0.076	1.3	M	Normal
34	0.073	1.3	M	Normal
1	0.072	1.3	M	Normal
47	0.072	1.3	M	Normal
38	0.071	1.3	M	Nasopharyngeal
21	0.069	2.7	F	Right breast invasive ductal carcinoma, appendicitis
14	0.067	2.6	F	Intrauterine pregnancy
48	0.064	1.1	M	Normal
40	0.063	1.1	M	Normal
12	0.061	1.1	M	Normal
29	0.058	2.3	F	After surgery for the right shoulder
42	0.056	1.0	M	Normal
20	0.054	1.0	M	Normal
39	0.053	0.9	M	Normal
37	0.044	0.8	M	Normal
6	0.042	0.8	M	Normal
3	0.040	0.7	M	Intertrochanteric fracture of the left femur
41	0.040	0.7	M	Normal
44	0.040	0.7	M	Normal
46	0.040	1.6	F	Normal
35	0.038	0.7	M	Normal
7	0.035	0.6	M	Normal
45	0.035	0.6	M	Normal
5	0.035	1.4	F	Abnormal ampulla of Vater (likely to be malignant)
13	0.022	0.8	F	Hemorrhoids and ovarian cysts, bronchial asthma
36	0.019	0.7	F	Osteoarthritis knees
N1	0.162	6.3	F	Non‐small‐cell lung cancer in the right lung
N2	0.040	0.7	M	Normal
N3	0.056	1.0	M	Normal
N4	0.081	1.4	M	Normal
N5	0.040	0.7	M	Normal
N6	0.035	0.6	M	Normal
N7	0.040	1.6	F	Normal
N8	0.072	1.3	M	Normal
N9	0.064	1.1	M	Normal

In another batch of genetic screening for nine subjects, we found that a female patient diagnosed with non‐small‐cell lung cancer had an absolute lifetime risk of 16.2% and a relative lifetime risk of 6.3 (Table 2), which was significantly higher than that of the remaining eight normal subjects. These results, although based on a test with a small group size and a limited number of positive patients, indicated that our algorithm was sensitive, to a certain degree, in screening the high‐risk lung cancer patient.

## Conclusions

In this study, we developed a general logistic model‐based algorithm to calculate the disease risk by directly using multiple SNP risk factors from multiple publications, and tested this algorithm on Chinese individuals for lung cancer risk prediction. The logistic algorithm that we explored was apparently more intuitive and self‐evident than the algorithms adopted by commercial DRP providers 7, 8, 9, 10, 11, although similar input parameters were used 9, 10 (i.e., the average population risk of disease, the OR value and the genotype and/or allele frequencies of the SNPs).

Our logistic algorithm had several advantages: (a) the coefficient β in the risk calculation equation directly reflected the contribution of each SNP and was simply determined by the OR value; (b) the SNP risk factor markers and the OR value were adjustable and/or promptly updated if necessary, such that β_0_ could be recalculated using the new set of SNP markers; (c) the SNP risk factor markers, with or without the OR value for the homozygous forms, were all suitable for risk prediction; and (d) accordingly, the non‐SNP risk factors (e.g., smoking and drinking) could also be included in our logistic model for DRP. Therefore, our study is of interest for the commercialization of DRP, given that similar logistic model‐based algorithms can be established for common complex diseases (e.g., diabetes, cardiovascular disease and cancers) that have been reported to be linked quantitatively to multiple SNP risk factors 17.

## Conflict of interest

The authors declare no conflict of interest.

## Author contributions

XF designed the research and established the model. GL performed genotyping experiments. XF and CL analyzed the data and wrote the manuscript.

## Supporting information


**Data S1.** DRP algorithms of commercial companies.

**Table S1.** SNP profiling results of 48 individuals.
