# Supplementary material for: Development of a general logistic model for disease risk prediction using multiple SNPs
Source: FEBS Open Bio. 2019 Sep 27;9(11):2006–12. doi: 10.1002/2211-5463.12722 (PMC6823278; doi:10.1002/2211-5463.12722)
Supplement: Supplementary file 1 — Data S1. DRP algorithms of commercial companies. [file FEB4-9-2006-s001.pdf]

Supporting information file

Original technical documents on the DRP algorithms used by three companies (i.e., 23andMe, Navigenics and deCODEme) were posted on their websites, but now are not available there. As reported earlier (1-4), researchers utilized these algorithms for DRP. In one of these reports (Kido T, et al. 2013. Systematic evaluation of personal genome services for Japanese individuals. J Hum Genet 58:734-741.), the principles of the algorithms were clearly presented in the supporting information file, from which the texts are directly cut and pasted below as supporting information for our study.

1. Ng PC, Murray SS, Levy S, Venter JC An agenda for personalized medicine. Nature 2009;461:724-6.
2. Kido T, Kawashima M, Nishino S, Swan M, Kamatani N, Butte AJ Systematic evaluation of personal genome services for Japanese individuals. J Hum Genet 2013;58:734-41.
3. Kalf RR, Mihaescu R, Kundu S, de Knijff P, Green RC, Janssens AC Variations in predicted risks in personal genome testing for common complex diseases. Genet Med 2014;16:85-91.
4. Kutz GD, Direct-to-consumer genetic tests: misleading test results are further complicated by deceptive marketing and other questionable practices, t.U.S.G.A. Office, Editor. 2010.

//

Supplemental Note 1. Risk prediction algorithms for 3 DTC companies.

We considered the case in which 1 biallelic marker was associated with a disease (denoted as a disease-associated locus). A disease-associated locus was assumed to consist of 2 alleles, a and A, the former resulting in an increased disease risk. Let p be the frequency of risk allele a, and assuming Hardy-Weinberg equilibrium, the frequency of each genotype AA, Aa, and aa can be represented by $(1-p)^2, 2p(1-p)$, and p^2, respectively. Let d_1, d_2, and d_3 be the penetrance (probability of developing the disease) of each of genotype AA, Aa, and aa.

We can represent the prevalence of the disease in the population (q) as follows:

$$q = d_1(1-p)^2 + 2d_2p(1-p) + d_3p^2$$

d_i denotes the probability that an individual with each genotype randomly selected from the population has the disease.

By using these notations, the risk prediction algorithm for the 3 companies (the method for calculating an individual's absolute risk with multi-allelic markers) can be described as follows.

23andMe

Let $\mathbf{r}^{(1)}$ and $\mathbf{r}^{(2)}$ be the OR defined by the following formulas:

$$\mathbf{r}^{(1)} = \frac{\frac{\mathbf{d_2}}{1-\mathbf{d_2}}}{\frac{\mathbf{d_1}}{1-\mathbf{d_1}}}$$

$$\mathbf{r}^{(2)} = \frac{\frac{\mathbf{d_3}}{1-d_3}}{\frac{d_1}{1-d_1}}$$

Therefore, if we are given q, p, $r^{(1)}$, and $r^{(2)}$, then we can get (d_1, d_2, and d_3) by solving the above 3 equations, since there are 3 equations with 3 variables.

Then, individual absolute risk with multi-allelic markers (P) is calculated by the following formulas.

$$x = \frac{q}{1-q}\prod_{j=1}^{n}\left(\frac{d_i g_i}{1-d_i g_i}\Big/\frac{q}{1-q}\right)$$

$$P = \frac{x}{1+x}$$

where n represents the number of allelic markers and d_ig_i represents penetrance when the i locus of an individual's genotype was g_i.

Navigenics

Navigenics uses the risk ratio, $\lambda_{RN} = d_2/d_1$, and $\lambda_{RR} = d_3/d_1$, instead of using d_1, d_2, d_3.

We can obtain λ_{RN} and λ_{RR} by solving the following 2 equations:

$$r^{(1)} = \lambda_{RN} \frac{q/d_1 - q}{q/d_1 - q\lambda_{RN}}$$

$$r^{(2)} = \lambda_{RR} \frac{q/d_1 - q}{q/d_1 - q\lambda_{RR}}$$

Then, individual absolute risk with multi-allelic markers (P), which is termed GCI (genetic composite index) by Navigenics, is calculated by the following formula:

$$P = GCI(g_1, g_2, \dots g_n) = \prod_{i=1}^{n} \lambda_{g_i}$$

where n represents the number of allelic markers, g_i represents the genotype of locus i, and λ_{gi} represents the risk ratio for the genotype (for genotype AA, Aa, and aa, λ_{gi} = 1, d_2/d_1, and d_3/d_1, respectively.)

deCODEme

deCODEme uses the odds ratio(OR) instead of the risk ratio. They assume that the OR can be approximated as the value of the risk ratio.

$$r' = \frac{d_2}{d_1} \cong \frac{d_2}{1 - d_2} \Big/ \frac{d_1}{1 - d_1}$$

$$r' = \frac{d_3}{d_2} \cong \frac{d_3}{1-d_3} \Big/ \frac{d_2}{1-d_2}$$

Since the prevalence of the disease in the population (q) can be described as follows:

$$q = d_1(1-p)^2 + 2d_2p\,(1-p) + d_3p^2,$$

the relative risk for each genotype aa, Aa, AA can be obtained by solving the above equations as follows:

$$\frac{d_3}{q} = \frac{d_1r'^2}{q} = r'^2/R$$

$$\frac{d_2}{q} = \frac{d_1r'}{q} = r'/R$$

$$\frac{d_1}{q} = 1/R$$

where, $R = (1-p)^2 + 2p\,(1-p)r' + d_3r'^2$.

Then, individual absolute risk with multi-allelic markers (P) is calculated by the following formulas.

$$\frac{P}{q} = \prod_{i=1}^{n}\left(\frac{d_{ig_i}}{q}\right)$$

where n represents the number of allelic markers, and d_ig_i represents penetrance when the i locus of an individual's genotype was g_i.
