# Supplementary material for: Development of a general logistic model for disease risk prediction using multiple SNPs
Source: FEBS Open Bio. 2019 Sep 27;9(11):2006–12. doi: 10.1002/2211-5463.12722 (PMC6823278; doi:10.1002/2211-5463.12722)
Supplement: Supplementary file 3 [file FEB4-9-2006-s003.docx]

Data S1:-

DRP algorithms of commercial companies.

Table S1:-

SNP profiling results of 48 individuals.
